# Molecular mechanism of paraquat-induced ferroptosis leading to pulmonary fibrosis mediated by Keap1/Nrf2 signaling pathway

**DOI:** 10.1007/s11033-023-08756-z

**Published:** 2023-10-09

**Authors:** Xiaoxia Yang, Ping Xiao, Xiaofeng Shi

**Affiliations:** 1 Department of Neurology, Tianjin First Central Hospital, Tianjin, 300192 China; 2 Clinical Laboratory, Tianjin First Central Hospital, Tianjin, 300192 China; 3 Department of Emergency, Tianjin First Central Hospital, Tianjin, 300192 China

**Keywords:** Ferroptosis, Paraquat, Pulmonary fibrosis, Keap1/Nrf2 signaling pathway, Molecular mechanism

## Abstract

Paraquat (PQ) is a widely used and highly toxic pesticide that is often actively ingested and causes pulmonary fibrosis in patients. Ferroptosis is a regulated form of non-apoptotic cell death associated with iron-dependent lipid peroxidation. Previous studies have shown that ferroptosis is involved in the occurrence and development of acute lung injury (ALI). In this study, a model rat with inflammatory response, oxidative stress, lipid peroxidation, and pulmonary fibrosis was successfully established by PQ administration. The occurrence of ferroptosis in PQ model rats was confirmed by TUNEL staining, iron ion detection, and Ferroptosis related biomarkers detection. Western blotting (WB) and real-time PCR (RT-PCR) showed that the expression of Keap1 was significantly up-regulated and the expression of Nrf2 was significantly down-regulated in the lung tissue of PQ rats. Further transcriptomics and proteomics confirmed: (1) Enrichment of molecular processes related to iron ion binding; (2) Keap1 may promote Nrf2 ubiquitination and lead to Nrf2 degradation; (3) There is functional enrichment in ferroptosis related pathways. Our results suggest that PQ can regulate Keap1/Nrf2 signaling pathway, leading to increased lipid peroxidation and abnormal iron uptake, thereby inducing iron death and exacerbating the progression of pulmonary fibrosis. Our study provides new insights into PQ-induced pulmonary fibrosis.

**Graphical abstract:**

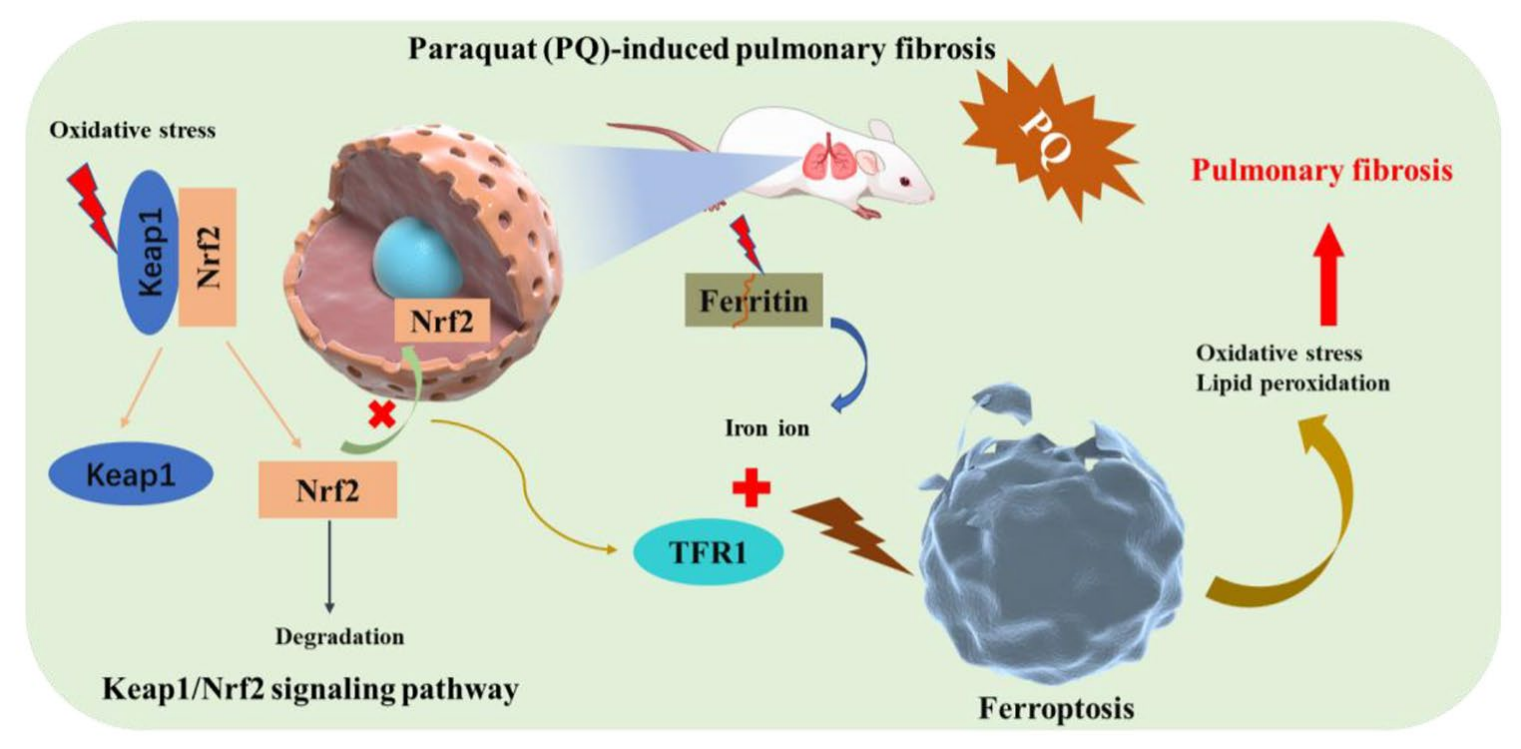

**Supplementary Information:**

The online version contains supplementary material available at 10.1007/s11033-023-08756-z.

## Introduction

Paraquat (PQ, 1,1′-dimethyl-4,4′-bipyridylium dichloride) has a guaranteed weed growth suppression effect and is widely used worldwide. Paraquat is 28 times more toxic than glyphosate, another common herbicide, and is trending toward replacing the latter in some countries and regions [1].Unfortunately, many people die from PQ poisoning each year globally, and accidental or intentional ingestion during suicide is a common cause of poisoning [2]. Currently, there is no specific antidote for PQ poisoning [3]. PQ is rapidly absorbed after ingestion. Even though about 90% of the absorbed PQ is excreted as a parent compound through the kidneys within the first 24 h [4]. However, PQ-induced kidney damage can lead to rapid deterioration of kidney function within the first few hours [5]. Eventually, PQ will accumulate selectively in the lungs in the polyamine uptake system, producing devastating damage in the target organ, the lung [6]. In addition, parent toxic molecules will replicate in redox reactions leading to the onset of lung cell cascade damage [7]. In the last decade, there have been increasing reports of PQ toxicity.However, the main molecular mechanisms of PQ-induced pulmonary toxicity have not been fully elucidated.

Ferroptosis is a special form of cell death, distinct from apoptosis, autophagy, and other forms of cell death, and its formation is characterized by abnormal aggregation of iron ions and the production of harmful free radicals [8, 9]. Mechanistically, eukaryotic cells are under constant attack by lipid peroxides produced by the peroxidation of phospholipids of polyunsaturated-fatty-acid-containing phospholipids (PUFA-PLs) in the cell membrane under conditions of iron enrichment and reactive oxygen species (ROS) [10, 11]. Without intervention, these toxic lipid peroxides can build up to lethal levels, destroying the integrity of cell membranes and eventually triggering siderophile cell death [12]. Ferroptosis is associated with a variety of diseases, including inflammation, neurodegenerative diseases, ischemia-reperfusion injury, brain injury, etc [13]. Studies have shown that ferroptosis is a possible therapeutic target for a variety of lung-related diseases, such as acute lung injury, pulmonary fibrosis, lung infections, chronic obstructive pulmonary disease, and asthma [14, 15]. Li et al. reported that ferroptosis inhibitors can alleviate pulmonary fibrosis, suggesting that ferroptosis may mediate the occurrence and development of pulmonary fibrosis [16]. Ferroptosis inhibitors with antioxidant properties are one of the potential compounds to overcome the toxicity of PQ. In addition, Ma et al. reported that regulating iron accumulation may be a potential strategy to inhibit pulmonary fibrosis [17]. Moreover, increased lipid peroxidation and oxidative stress are critical to the progression of PQ poisoning. The pathogenesis of PQ toxicity is related to early acute lung injury and late pulmonary fibrosis, and oxidative stress and inflammation may be involved in this process [18]. However, whether ROS accumulation and the presence of lipid peroxidation in PQ poisoning induce ferroptosis and the potential role of Ferroptosis in tissue fibrosis induced by PQ poisoning still face a knowledge gap.

Nuclear factor erythroid 2-related factor 2 (Nrf2) is a crucial transcription factor in cellular antioxidant response and a major ferroptosis signaling molecule [19]. In general, Nrf2 binds to Kelch-like ECH-associated protein 1 (Keap1) and is thus localized in the cytoplasm. Under oxidative stress, however, Nrf2 mediates the detoxification process by dissociating from Keap1, which is then transported to the nucleus and activates the Nrf2 transcription gene [20]. Activation of Nrf2 reduces iron absorption, limits ROS production, and enhances the antioxidant capacity of cells [8]. Therefore, Nrf2 can inhibit the ferroptosis. In addition, the activated Keap1/Nrf2 signaling pathway regulates the expression of a range of antioxidant and cellular protection genes, such as glutathione reductase, peroxidase, and heat shock protein [21]. The enhanced expression of these genes can increase the resistance of cells to free radical damage and promote cell repair and recovery. However, the regulation of Keap1/Nrf2 signaling pathway by PQ poisoning and the molecular mechanism of whether Keap1/Nrf2 signaling pathway induces Ferroptosis and leads to pulmonary fibrosis is still lacking.

In this study, we established a PQ-poisoning model by administering PQ to rats. The process of pulmonary fibrosis in model rats was monitored, and the expression levels of Collagen II, α-SMA, and Fibronectin markers in lung tissue were detected by real-time quantitative fluorescent PCR (qRT-PCR) and Western blotting (WB). The content of malondialdehyde (MDA) in the lungs was detected to evaluate the lipid peroxidation level of lung tissue. The expression of Keap1 and Nrf2 genes related to the Keap1/Nrf2 signaling pathway in the lung of model rats was detected by WB to investigate the effect of PQ poisoning on the Keap1/Nrf2 signaling pathway. The expression of ferroptosis related proteins PTGS2, NOX1, FTH1, COX2, GPX4, and ACSL4 in model rats was detected by WB to confirm whether PQ poisoning induced ferroptosis. The iron content in alveolar lavage fluid and serum of rats was detected to confirm the occurrence of iron accumulation. The contents of transferrin and ferritin in alveolar lavage fluid and serum were determined by enzyme-linked immunosorbent assay (ELISA). Finally, transcriptomics and proteomics were combined to explore the molecular mechanism of PQ poisoning regulating the Keap1/Nrf2 signaling pathway inducing ferroptosis leading to pulmonary fibrosis. This study provides data support for the potential molecular mechanism of PQ poisoning and potential screening targets for the treatment of pulmonary fibrosis caused by PQ poisoning.

## Materials and methods

### Experimental animals

A total of 27 SPF male SD rats were selected (Purchased from Beijing Vital River Laboratory Animal Technology Co., LTD., Beijing, China), 8–10 weeks old, 280–320 g. The rats were cultured in a suitable environment (Room temperature (22 ± 0.5℃), humidity 50 ± 5%, light/dark cycle 14 h/10 h). And incubate for a week before the experiment to acclimate to the laboratory environment. All experimental research processes are carried out in accordance with the experimental animal practice code and approved by the hospital’s Animal Research Committee (Approval No.: IACUC of AMMS-08-2021-019).

### Animal grouping and experimental procedure

Twenty-seven rats were randomly divided into control group (FC), PQ low-dose group (FL) and PQ high-dose group (FH) according to random number table. The FL and FH groups were administrated with PQ of 10 mg/kg and 50 mg/kg [22], respectively, while the FC group was administrated with the same amount of distilled water. On day 3 after PQ administration, intraperitoneal injections of ketamine (70 mg/kg, Sigma, San Francisco, CA, USA) and xylazine (10 mg/kg; Sigma) induced anesthesia and killed the rats. Whole blood samples of rats were collected and put into anticoagulant tubes for absolute cell count, and into pro-coagulant tubes for analysis of iron ion, transferrin and ferritin content, and analysis of inflammatory factors content. Take the lung and calculate the lung system. Subsequently, the left lobe was used for malondialdehyde (MDA), glutathione (GSH), Sirius red staining and transmission electron microscopy (TEM, FEI Talos F200x, USA) observation. RT-PCR and Western blot analysis were performed on the right upper lobe, histopathological analysis and iron determination on the right middle lobe, and transcriptomic and proteomic analysis on the right lower lobe.

### Histopathological analysis and sirian red staining

The isolated lung specimens were fixed with 10% formalin for 24 h before paraffin embedding. The paraffin blocks were cut into 4 μm thick slices using a microtome, dewaxed with xylene solution, and hydrated with 100, 90, 80, and 75 ethanol gradients in series. Morphological changes were examined using Sirius red staining according to the standard protocol [23].

### Transmission electron microscope observation

Lung tissue pieces were collected and fixed at 4 °C for 12 h using 2.5% glutaraldehyde. The lung tissue was then immobilized with 1% osmic acid for 1 h, dehydrated with graded ethanol and acetone, and then embedded in epoxy resin. The ultrathin sections were cut by Leica EM UC7 and the ultrastructural changes were observed under TEM.

### Immunofluorescence measurement

Use Collage I staining solution (EPR24331-53, 1:1000; Abcam) or α-SMA staining solution (Ready to use; Zsbg-bio, China) soaked frozen slides and placed in the dark at 37 °C for 30 min. After 3 washes, the slides were treated with DAPI solution (G1012; Servicebio) Let stand in the dark at room temperature for 10 min. Each tissue section was viewed under a microscope and images were collected through a fluorescence microscope.

### Detection of ROS, MDA and GSH

The commercial Reactive Oxygen Species Kit (Beyotime, China) was used to detect ROS levels in lung tissue.MDA and GSH levels in lung tissue were measured using a colorimetric biochemical kit (Nanjing Institute of Biological Engineering, Nanjing, China) in strict accordance with the manufacturer’s instructions.

### Inflammatory factor

IL-1β, IL-6, MCP-1, and TNF-α were determined by enzyme-linked immunoassay kit (ELISA). IL-1β (SEKR-0024), IL-6 (SEKR-0005), MCP-1 (SEKR-0024), and TNF-α (SEKR-0009) were all used rat ELISA kits from Solarbio Science and Technology Co., LTD., Beijing, China. The measurement of inflammatory factors was carried out in strict accordance with the manufacturer’s instructions.

### Analysis of iron content

The lung tissue and serum solution were dissolved using sulfuric acid: nitric acid (3:1), and the iron content of the dissolved solution was determined by o-phenanthroline colorimetry.

### TUNEL assay

The TransDetect® in Situ fluorescein TUNEL apoptosis detection kit is used to analyze apoptosis in strict accordance with the manufacturer’s protocol.

### Western blotting

The collected rat lung tissue samples were homogenized and ground with appropriate amounts of RIPA lysate (Solarbio, China) supplemented with protease inhibitors. After centrifugation at 4℃ at 8000 g for 10 min, the supernatant was collected and the total protein concentration was determined by BCA protein detection kit (Solarbio, China). The proteins were then isolated with 10% SDS-PAGE for 2 h, then transferred to a PVDF membrane (Millipore, USA) and sealed with 5% buttermilk for 2 h. The first anti GPX4 (1:1000), TRF1 (1:1000), DMT1 (1:1000), FTL (1:1000), Nrf2(1:1000), Keap1 (1:1000),p-GSK-3β (1:1000), Fyn (1:1000)and Nqo1 (1:1000) were purchased from Abcam, UK. The primary antibody was incubated at 37℃ for 12 h. Subsequently, PVDF membrane was washed with TBST buffer several times and incubated with two antibodies at room temperature for 1 h. Sheep Anti-Rabbit IgG-HRP (1:3000) and Sheep Anti-Mouse IgG-HRP (1:3000) were purchased from Bioss(China). After ECL color development, the automatic chemiluminescence image analysis system was used to determine the protein bands, and the gray values of the protein bands were analyzed by Gel-pro analyzer (Tanon, China) software.

### Real-time fluorescence quantitative PCR

Total RNA was extracted from rat lung tissue using Trizol reagent (Invitrogen, USA). The total RNA was then reverse-transcribed into cDNA using a cDNA synthesis kit (Thermo, USA). Next, RT-qPCR was performed using SYBR®Green kit (TaKaRa, Japan), and GAPDH was selected as the internal reference gene. Any operation shall be carried out in strict accordance with the supplier’s instructions. In addition, the primers used are given in Table S1.

### Transcriptomic analysis

Transcriptomic analysis methods are presented in the ***Supporting Information*** (***SI***) **Method S1**.

### Proteomic analysis

The proteomic analysis method is given in **SI Method S2**.

### Statistical analysis

All animal experiments were repeated with at least 6 parallel samples. The obtained experimental data are expressed in the form of mean ± SD. IBM SPSS 25.0 was used for statistical analysis, and independent sample t test was used for comparison between the two groups. p < 0.05 was considered statistically significant. Compared with the control group (FC), * p < 0.05, ** p < 0.01 and *** p < 0.001, respectively.

## Results

### Pulmonary fibrosis and pathological features induced by PQ poisoning

**Fig. 1:**
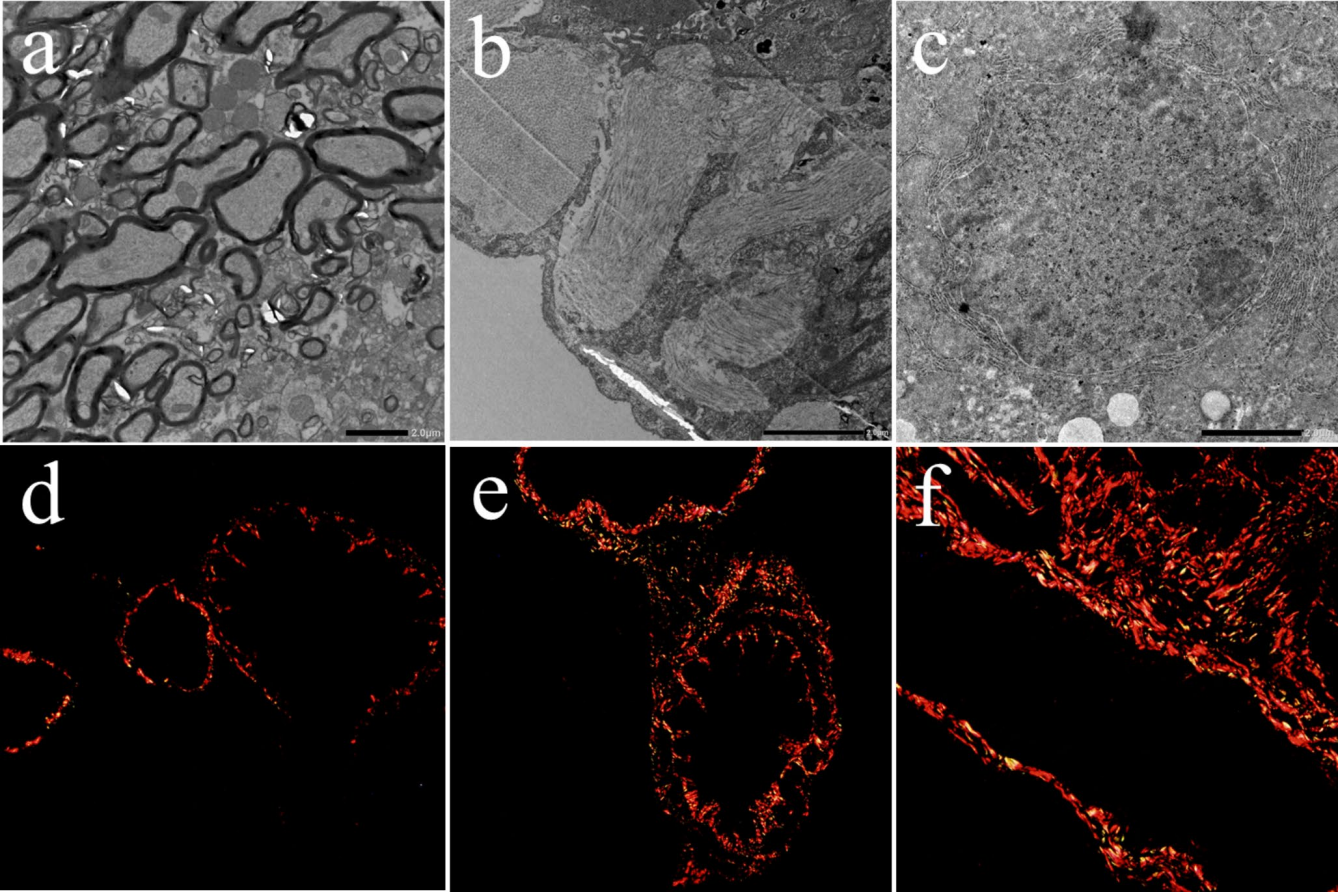
Pathological observation of rat lung biopsy (n = 6). TEM observation of **(a)** FC, **(b)** FL and **(c)** FH; Collagen fibers in lung tissue of rats in (a) FC, (b) FL and (c) FH groups (Sirius red staining, ×100)

The pulmonary histopathological changes of paraquat poisoning rats were observed by TEM. The results showed that the lung tissue structure of the FC group was complete, the alveolar shape and size were normal, the interval was clear, and there was no obvious inflammatory cell infiltration or fibrosis hyperplasia. After PQ modeling, the lung tissue structure of the rats with different degrees of damage and severe pulmonary fibrosis could be seen under the microscope (Fig. 1a-c). The results of Sirius red staining showed that no red collagen fibers were found in the interstitial lung of FC group mice (Fig. 1d). Compared with the FC group, the normal lung tissue structure of rats in each paraquat dose group was damaged, and there was a large amount of red collagen fiber deposition under the microscope, and the collagen fiber deposition was positively correlated with the paraquat dosage (Fig. 1e and f).Increased lung quotiety indicates edema of lung tissue, which is attributed to inflammation and tissue edema (Fig. S1).

**Fig. 2:**
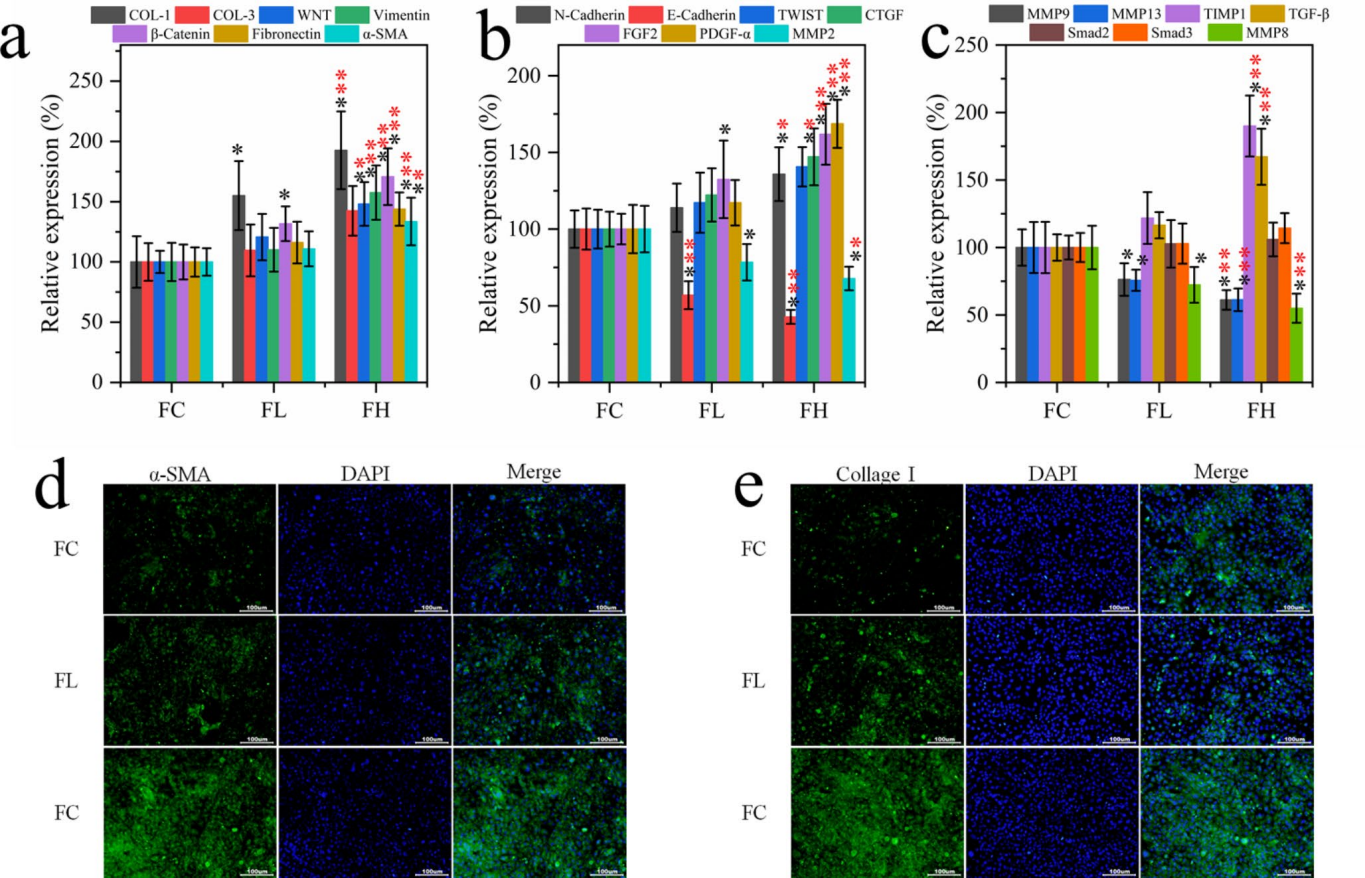
**(a-c)** ELISA was used to detect the expression level of fibrosis markers in lung tissue (n = 6). Immunofluorescence staining of **(d)** α-SMA and **(e)** Collagen 1, 100X, (n = 6). Symbol system: *p < 0.05; **p < 0.01and ***p < 0.001, respectively

**Fig. 3:**
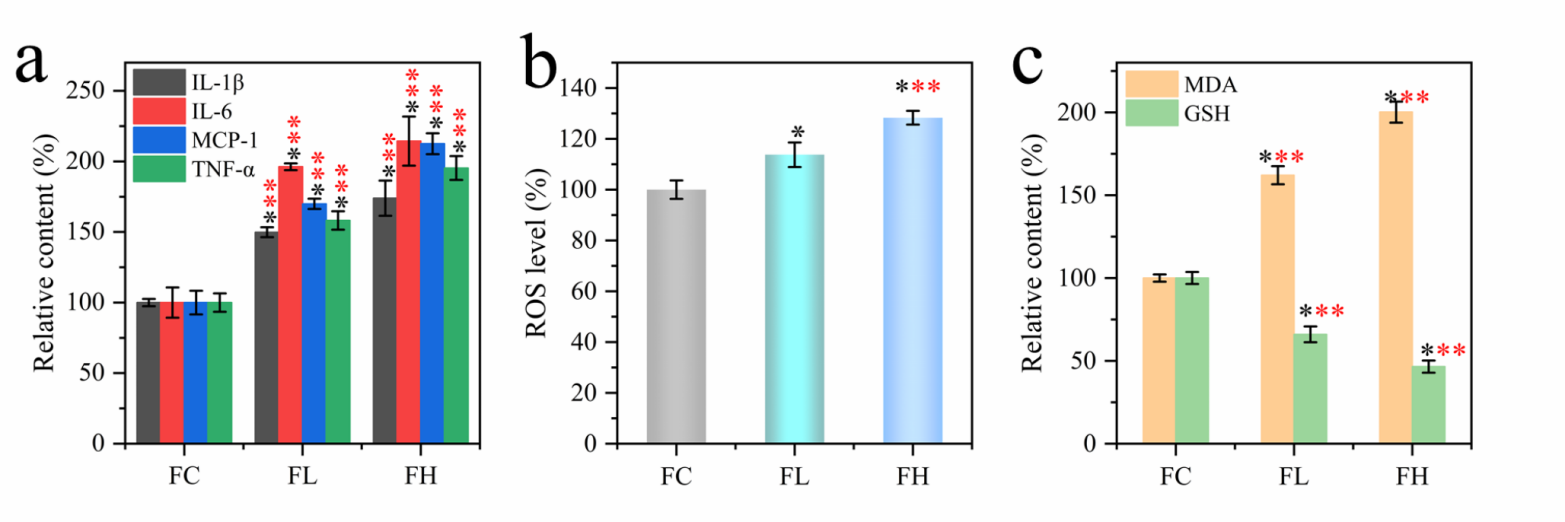
**(a)** The content of proinflammatory cytokines in blood of rats after PQ modeling (n = 6). **(b)** The level of reactive oxygen species (ROS) in lung tissues of rats in each group (n = 6). **(c)** Effects of PQ modeling on lipid peroxidation in rats: changes in MDA and GSH contents (n = 6).*p < 0.05; **p < 0.01and ***p < 0.001, respectively

Furthermore, ELISA was used to detect pulmonary fibrosis markers COL-1, COL-3, WNT, Vimentin, β-Catenin, Fibronectin, α-SMA, N-Cadherin, E-Cadherin, TWIST, CTGF, FGF2, PDGF-α, MMP2, MMP8, MMP9, MMP13, TIMP1, TGF-β, Smad2, and Smad3 expression levels (Fig. 2a-c). The results showed that compared with FC group, the expression levels of intermediate mesenchymal character-related proteins a-SMA and Fibronectin were significantly increased in PQ modeling groups (FC and FH), while the expression levels of epithelial character-related proteins such as E-cadherin and MMP family proteins were significantly decreased. At the same time, the protein contents of Vimentin, CTGF, FGF2, PDGF-α, TIMP-1, and TGF-β were significantly increased (p < 0.001). This suggests that PQ promotes epithelial interstitial transformation, which leads to the deposition of extracellular matrix protein in lung interstitial tissue, thereby causing lung tissue structural damage and pulmonary fibrosis. Immunofluorescence detection of epithelial cell to mesenchymal transformation (EMT) related protein expression results (Fig. 3d and e) showed that the expression levels of α-SAM and Collagen 1 proteins in the lung tissues of rats were increased after PQ modeling. This indicated that PQ modeling was successful, and the rat developed pulmonary fibrosis.

### PQ-induced inflammation and oxidative stress

It is known that PQ modeling causes pulmonary fibrosis in rats, so it is necessary to investigate the inflammatory response, oxidative stress, and lipid peroxidation levels caused by PQ poisoning. Herein, serum levels of proinflammatory cytokines IL-1β, IL-6, MCP-1, and TNF-α were measured in rats of each experimental group (Fig. 3a). The results showed that even low-dose PQ modeling resulted in significant upregulation of inflammatory cytokines in the model rats (p < 0.001). Among the four inflammatory factors, the up-regulation of IL-6 and MCP-1 were the most obvious, which were 196.2-214.4% and 169.9-212.5%, respectively. The significant increase of inflammatory factors indicated that PQ modeling induced inflammatory response in rats. Next, reactive oxygen species (ROS) in lung tissue were examined. As shown in Fig. 3b, ROS in lung tissue of rats increased significantly after PQ modeling (up 13.7–28.3%, p < 0.05). These results indicate that PQ poisoning can induce ROS increase in lung tissue. At the same time, malondialdehyde (MDA), the end product of lipid peroxidation, was detected (Fig. 3c). The results showed that PQ modeling induced the accumulation of lipid peroxidation products, indicating that PQ poisoning was accompanied by severe lipid peroxidation. Finally, the content of glutathione (GSH) was measured. The results showed that PQ modeling caused a serious deficiency of GSH content in rat lung tissue. As an important antioxidant, the depletion of GSH will lead to the loss of the body’s regulation of oxidative stress. The results showed that PQ modeling could induce inflammation and lead to oxidative stress and lipid peroxidation in rats.

### Ferroptosis induced by PQ exposure in rats

**Fig. 4:**
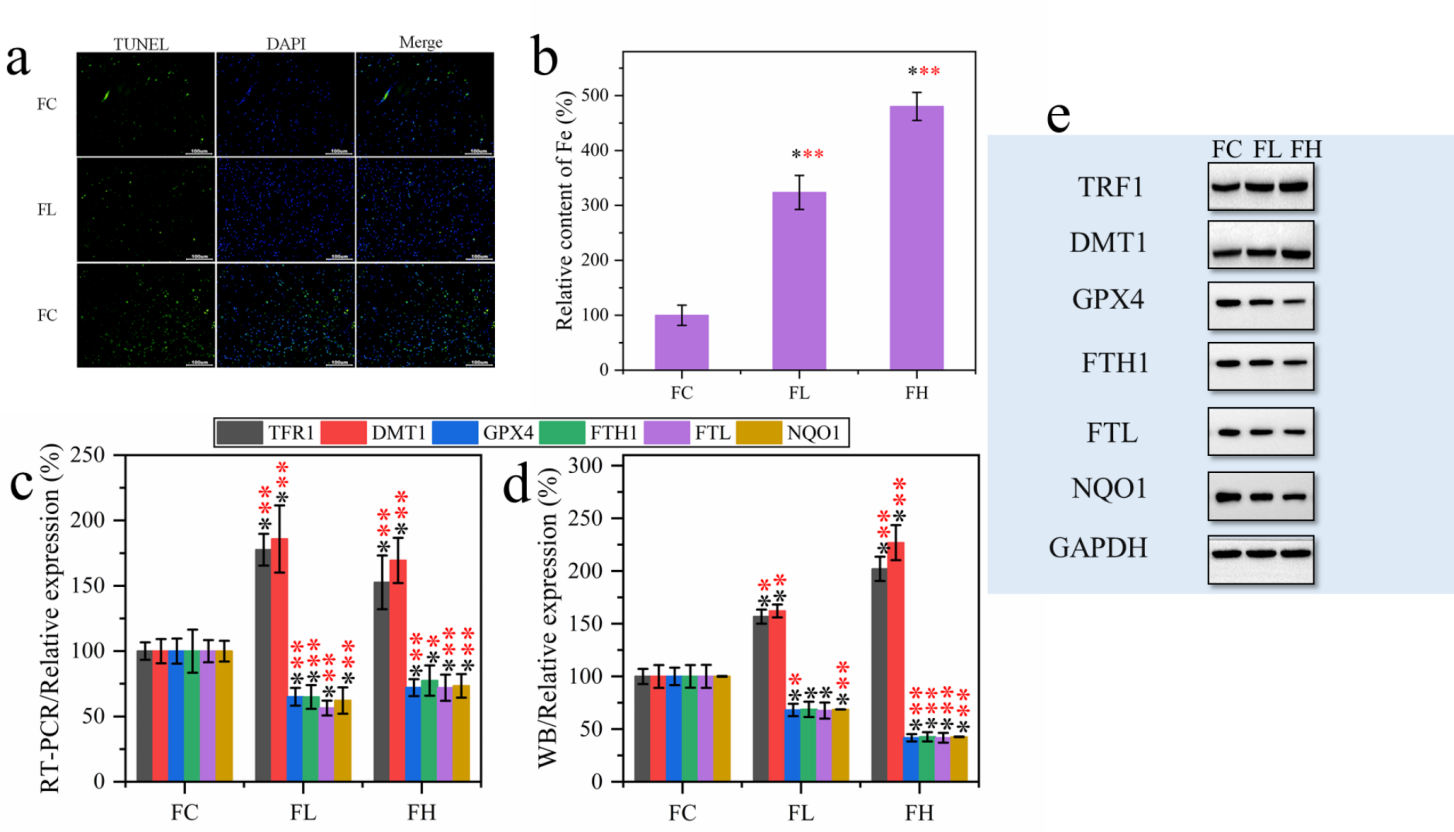
Ferroptosiswas induced by PQ modeling in rat lung cells (n = 6). **(a)** TUNEL dyeing. **(b)** Determination of iron content in lung tissue. **(c)** Effect of PQ modeling on Ferroptosis-related biomarker expression in rat lung tissue. (d, e) Western blotting (WB) determination of ferroptosis-related biomarker content statistics and WB bands.*p < 0.05; **p < 0.01and ***p < 0.001, respectively

Ferroptosis is a new type of iron-dependent programmed cell death. Herein, we show that PQ modeling induces apoptosis in rat lung tissue by TUNEL staining (Fig. 4a). To determine whether this was attributable to ferroptosis, the amount of Fe in the lung tissue of each group of rats was measured (Fig. 4b). The results showed that Fe content in lung tissue of PQ modeling rats increased significantly (p < 0.001) and was 3.2–4.8 times that of normal rats. Cell uptake of Fe is disturbed, which will lead to oxidative stress and the appearance of lipid peroxidation, as shown in the results in the previous section. Next, ferroptosis related biomarkers such as TFR1, DMT1, GPX4, FTH1/FTL and NQO1 were detected (Fig. 4c-e). In Fig. 4c, the expression of several biomarker related genes detected by RT-PCR showed that PQ modeling significantly up-regulated the expression of TFR1 and DMT1 (p < 0.001), while down-regulated the expression of GPX4, FTH1/FTL, and NQO1 (p < 0.001). Furthermore, the expression products of related biomarker genes were detected and a consistent rule was obtained (Fig. 4d and e). Therefore, we suggest that the modeling of PQ leads to programmed cell death, and this programmed death pattern can be attributed to ferroptosis.

### Response of Keap1/Nrf2 signaling pathway to PQ poisoning

**Fig. 5:**
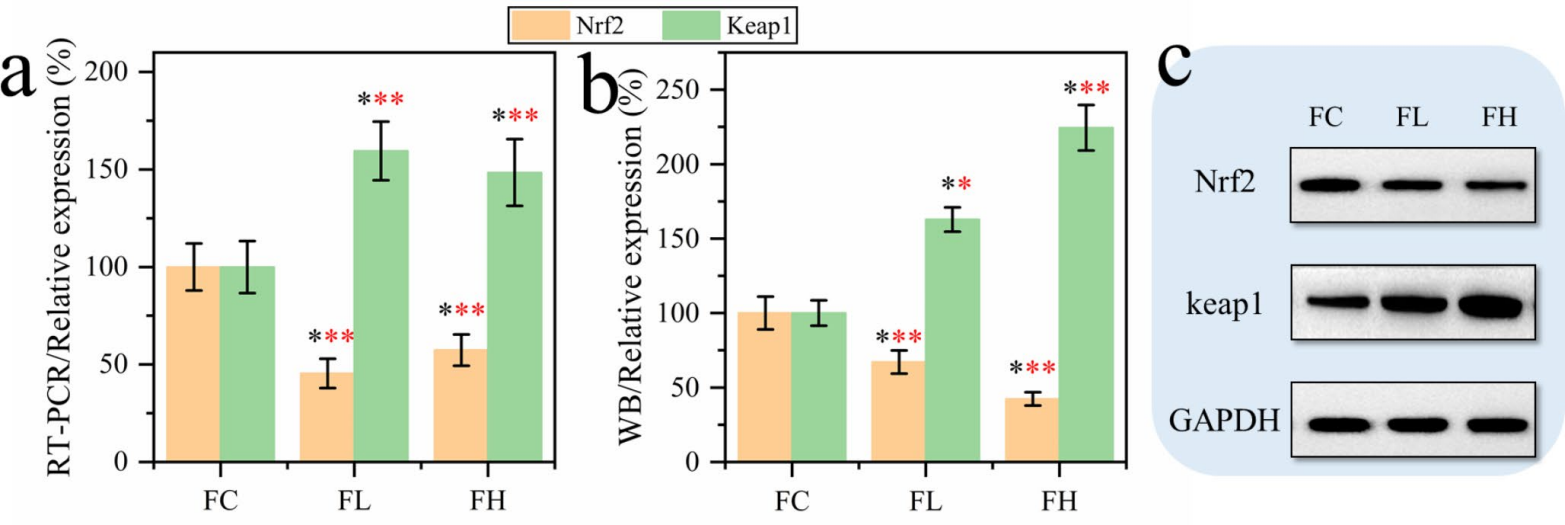
Regulation of Keap1/Nrf2 signaling pathway in rat lung by PQ modeling (n = 6). **(a)** Effect of PQ modeling on Keap1/Nrf2 related gene expression in rat lung tissue. **(b, c)** Western blotting (WB) analysis of Keap1/Nrf2 protein content and WB bands (n = 6).*p < 0.05; **p < 0.01 and ***p < 0.001, respectively

Keap1/Nrf2 signaling pathway is considered to be one of the vital pathways involved in the regulation of oxidative stress [24–26]. Its role in the regulation of oxidative stress may be a bridge to the pathogenesis of pulmonary fibrosis and ferroptosis caused by PQ poisoning. Therefore, it is necessary to investigate the changes of Keap1 and Nrf2 contents in the lung tissues of rats after PQ modeling. Here, we tested Keap1 and Nrf2 genes and their expression products (Fig. 5). The results showed that PQ could significantly down-regulate the expression of Nrf2 (p < 0.01) and up-regulate the expression of Keap1 (p < 0.01). This suggests that the Keap1/Nrf2 signaling pathway responds in PQ modeling, suggesting a critical role for this signaling pathway in investigating the relationship between ferroptosis and lung tissue fibrosis.

### Transcriptomic analysis of the effects of PQ exposure

**Fig. 6:**
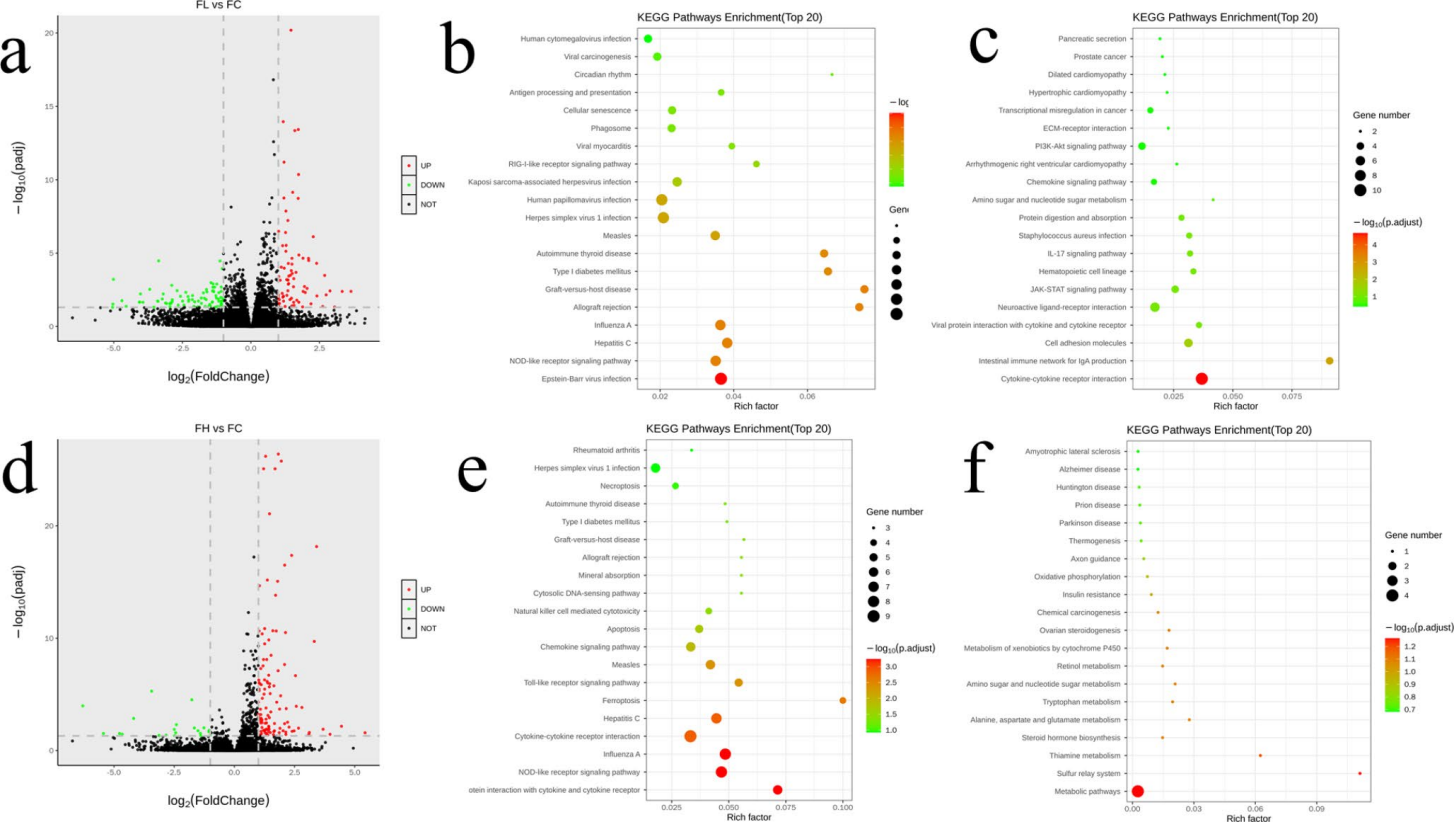
The number of **(a, d)** differential genes, the up-regulated **(b, e)** differential genes and the down-regulated **(c, f)** differential genes in the FL vs. FC and FH vs. FC groups

Transcriptomics was used to understand the potential role of genes in PQ-induced pulmonary fibrosis at the gene level. Herein, we found that increasing the dose of PQ decreased the number of differential genes, and the number of differential genes was the lowest among the PQ-exposed groups (Fig. 6a and d, and S3). This indicates that the potential genes affected by PQ will be concentrated with the increase of PQ concentration, and the differential genes exposed to high doses of PQ can be considered as the main response genes. Next, KEGG enrichment pathway analysis was performed for differential genes in the high-low dose PQ exposure group. In the up-regulated KEGG pathway, the high and low dose groups mainly concentrated in the process of viral infection, while the high dose group also produced KEGG pathway enrichment during ferroptosis (Fig. 6b and e). This provides direct evidence that high doses of PQ exposure induce ferroptosis at the genetic level. In the down-regulated KEGG pathway, cytokine-cytokine receptor interactions were observed as the dominant KEGG enrichment pathway in the low-dose group, while enrichment in the metabolic pathway was observed in the high-dose group (Fig. 6c and f).

### Proteomic analysis of the effects of PQ exposure

Through proteomics, the potential protein-binding sites of lung fibrosis induced by ferroptosis induced by PQ inhibition of the Keap1/Nrf2 signaling pathway were investigated. Herein, we identified a total of 7280 proteins (**Fig. S5a**). Compared with FH group, FL group showed more differential proteins, which was similar to the results of transcribing differential genes. Meanwhile, it was noted that upregulated proteins accounted for a higher proportion of differential proteins in the FH group (**Fig. S5b**), indicating that the toxic effect of PQ induced the expression of some proteins. Next, the protein domains detected between different groups were analyzed (**Fig. S6**). The results showed that low-dose PQ exposure mainly affected the immunoglobulin V-set domain (**Fig. S6a** and **S6b**), while high-dose PQ exposure mainly affected the trypsin domain and enriched the HMG14/HMG17 domain (**Fig. S6c** and **S6d**). This suggests that dose affects the binding target of PQ to the protein.

**Fig. 7:**
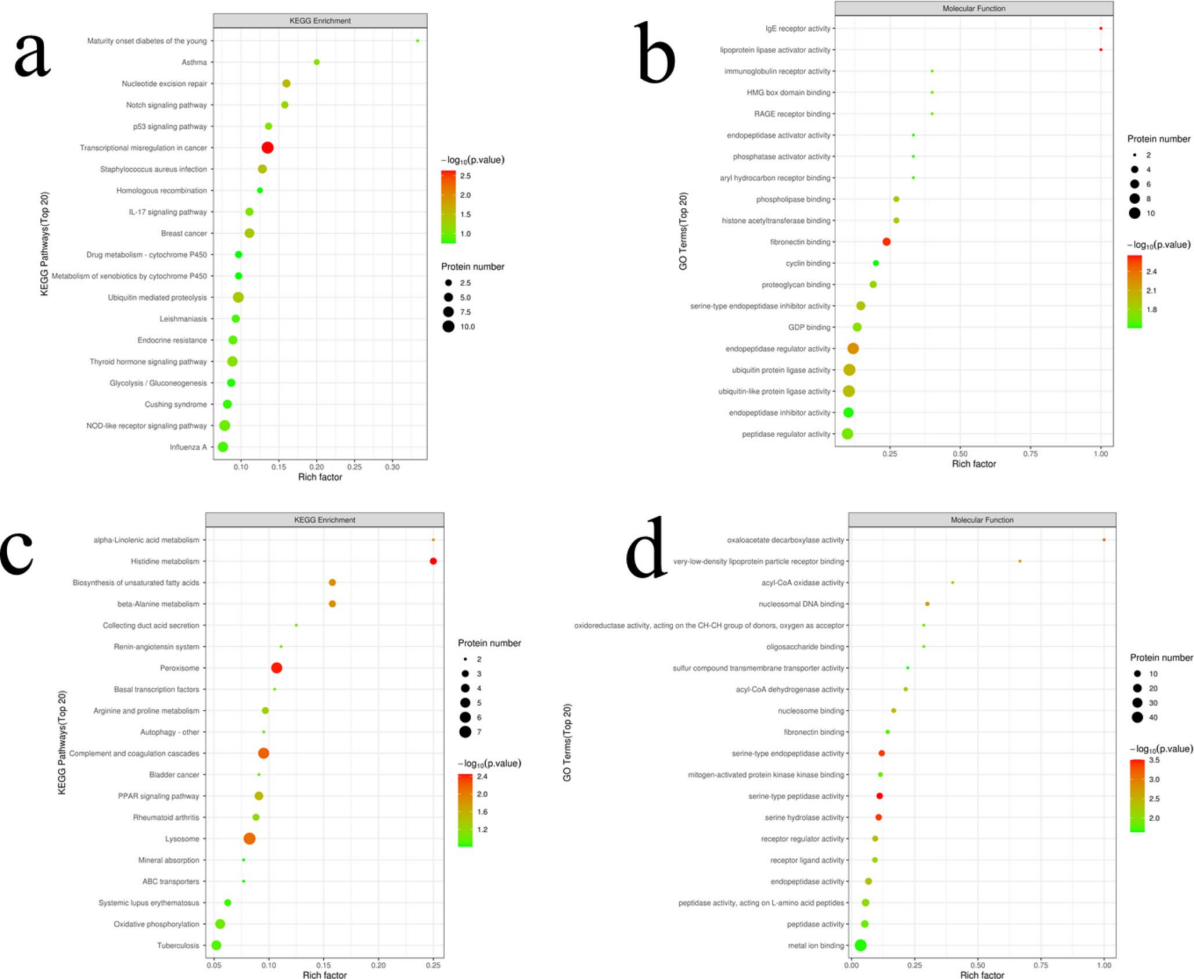
Functional analysis based on proteomics. **(a, c)** KEGG analysis of FL vs. FC and FH vs. FC. **(b, d)** KEGG analysis of FL vs. FC and FH vs. FC.

Next, the potential functional protein classes that respond to PQ exposure were identified (Fig. 7). The results showed that low-dose PQ exposure resulted in significant enrichment of the KEGG pathway, a transcription disorder in cancer (Fig. 7a). In addition, fibronectin binding sites, ubiquitin-protein ligase activity, and endopeptidase regulator activity are enriched GO pathways (Fig. 7b). At high doses of PQ, peroxidase and lysosome were the major KEGG enrichment pathways (Fig. 7c), and metal ion binding was the GO-enrichment pathway containing the largest number of proteins (Fig. 7d). This may be attributed to the fact that low doses of PQ have a carcinogenic risk and promote the metastasis of active cells, while high doses of PQ induce ROS and induce potential metal ion (Fe) enrichment [27, 28].

## Discussion

Acute lung injury or acute respiratory distress syndrome caused by paraquat (PQ) poisoning, followed by interval and pulmonary interstitial fibrosis, high mortality, most patients are associated with active drug suicide [29, 30]. However, there is still no effective treatment or specific antidote for PQ poisoning [31]. Thus, finding the pathogenesis or target of PQ exposure will help to understand the toxic mechanism of PQ and help people to design and develop effective detoxification therapies [14]. In this study, we established a rat model of pulmonary fibrosis by paraquat intragastric administration. The model showed inflammatory infiltration, oxidative stress, lipid peroxidation, and pulmonary interstitial fibrosis [32]. During the investigation of programmed cell death, iron accumulation and the occurrence of ferroptosis were found. PQ modeling has a significant effect on the expression of the Keap1/Nrf2 signaling pathway in rat lung tissue. In addition, proteomic and transcriptomic studies have been conducted to further explore the possible pathogenesis sites and mechanisms. Therefore, this study provides a new reference for further understanding of the mechanism of PQ toxicity.

Ferroptosis is a newly discovered mode of programmed cell death, which is different from apoptosis and autophagy and is triggered by Fe^2+^-dependent lipid peroxidation [33]. Herein, we found programmed cell death in lung tissue of PQ-poisoned model rats (Fig. 4a). The detection of Fe in lung tissue found that the uptake of Fe in lung tissue cells was seriously disturbed, and the concentration of Fe in normal tissue was 3–5 times. A high concentration of Fe can easily be used as a catalyst to induce Fenton reaction to produce reactive oxygen species including ^·^OH [34]. In addition, Fe binding to cell membrane can induce peroxidation damage of lipids on the cell membrane. As a result, oxidative stress and lipid peroxidation occur in cells. Our results confirm that lipid peroxidation and ROS content increase in PQ model rats. However, the available evidence is still insufficient to support a definitive link between ferroptosis and PQ poisoning. Therefore, follow-up transcriptomic and proteomic tests were performed. Among the top 20 metabolically enriched KEGG pathways, iron death was observed in the FH group as the most significantly enriched upregulated KEGG pathway (Fig. 6e). In addition, receptor interactions between cytokines and cytokines were observed to be the most significantly enriched down-regulated KEGG pathway (Fig. 6f). This indicates that PQ poisoning may lead to the loss of the body’s regulatory function of cytokines, which explains the reason for the accumulation of Fe in the lung tissue of PQ poisoning rats. At the same time, the analysis of proteomics shows that metal ion binding is the molecular process with the highest protein content. Therefore, it can be considered that PQ poisoning can cause serious disturbance of the molecular process of protein binding to Fe ions [35]. In other words, after PQ poisoning, Fe ions are overtaken by targeted proteins in the lung tissue, and the overtaken Fe^2+^/Fe^3+^ acts as an exogenous catalyst to induce reactive oxygen species and lipid peroxidation at the binding site (cell membrane), and finally ferroptosis is induced. Moreover, the progression of pulmonary fibrosis in PQ-poisoned rats was aggravated.

Furthermore, the detection of ferroptosis-related biomarkers showed that there was upregulation of transferrin receptor protein 1 (TFR1) anddivalent metal transporter 1 (DMT1). This suggests that the enriched iron in lung tissue may be taken up Fe^3+^via transferrin (TF) and TFR1, and theFe^3+^ is reducedand then transported to labile iron pool (LIP) via DMT1 [36–38].In addition, glutathione peroxidase 4 (GPX4), ferritin heavy chain (FTH1) and ferritin light chain (FTL) were down-regulated (Fig. 4c-e). Under normal conditions, iron is stably stored in cells in the form of ferritin (which consists of FTH, which acts as iron reductase, and FTL, which stores large amounts of iron) [39]. By using GSH, GPX4 can transform the cytotoxic lipid peroxides (L-OOH) of lipid peroxidation into the corresponding alcohols (L-OH), losing its peroxide activity, and this process acts as a resistance to ferroptosis of cells [40, 41]. Therefore, we speculate that PQ poisoning mediates the inactivation of iron chelating agent and lipophilic antioxidant inhibition. That is, PQ poisoning causes the Fe anchored by FTH1/FTL to be shed, which in turn is taken up and transported into the LIP by TFR1 and DMT1. Due to the inactivation of oxidative stress inhibitors, Fe^2+^ is transferred from the LIP and into lung tissue cells, inducing ferroptosis under the action of various factors. Next, ferroptosis further promoted inflammatory response, lipid peroxidation, and oxidative stress, leading to the intensification of the pulmonary fibrosis process.

Transcription factor nuclear factor erythroid 2-related factor 2 (Nrf2), a key regulator of cellular antioxidant responses, is thought to play a critical role in antioxidant stress, lipid peroxidation, and ferroptosis [42]. In this study, we found that the content of Nrf2 in lung tissue was significantly down-regulated, while the content of Kelch-like ECH-associated protein1(Keap1) was significantly up-regulated. Normally, Keap1 interacts with Nrf2 to promote polyubiquitination of Nrf2 proteins [43]. In addition, the Keap1 protein binds to the Nrf2 protein, iron ions are located in the cytoplasm. When a state of oxidative stress occurs, the binding of the Keap1 protein to Nrf2 is disturbed, causing the Nrf2 protein to be released from the Keap1 protein and enter the nucleus. The activation of Nrf2 in the nucleus can inhibit the expression of iron transporter. In the functional analysis of proteome, we found that the molecular processes involved in ubiquitination-like protein ligase activity and ubiquitin-protein ligase activity are enriched, and the molecular processes are the ones with a large number of proteins (Fig. 7b). These results indicated that PQ poisoning regulated Keap1 expression, and then promoted Nrf2 two-point polyubiquitination, leading to Nrf2 degradation [8]. Our results show that Nrf2 expression is significantly down-regulated in PQ-modelled rats, resulting in loss of cell inhibition of iron transporter expression. ferroptosis is induced by uncontrolled iron in lung tissue cells combined with oxidative stress, lipid peroxidation, and inflammation. In turn, the occurrence of ferroptosis further intensifies oxidative stress, lipid peroxidation, and inflammatory reactions, which will accelerate the process of pulmonary fibrosis.

## Conclusion

In summary, our findings reveal a possible molecular mechanism for the progression of pulmonary fibrosis induced by PQ poisoning. First, PQ poisoning regulates the Keap1/Nrf2 signaling pathway by promoting the expression of Keap1 in lung tissue, thus promoting Nrf2 degradation. This leads to the activation of transferrin (TFR1) and the occurrence of oxidative stress. The activity of transferrin is enhanced and the breakdown of ferritin leads to the stripping and transfer of anchored Fe ions. Furthermore, ferroptosis is induced by the abnormal accumulation of iron, inflammation, oxidative stress, lipid peroxidation, and other factors. The appearance of Ferroptosis further aggravates oxidative stress and lipid peroxidation, thus leading to the intensification of the pulmonary fibrosis process.

### Electronic supplementary material

Below is the link to the electronic supplementary material.

Supplementary Material 1

Supplementary Material 2

## Data Availability

The authors declared that all relevant data and materials were available within the paper on reasonable request.
