# Supplementary material for: Molecular mechanism of paraquat-induced ferroptosis leading to pulmonary fibrosis mediated by Keap1/Nrf2 signaling pathway
Source: Mol Biol Rep. 2023 Oct 9;50(11):9249–61. doi: 10.1007/s11033-023-08756-z (PMC10635988; doi:10.1007/s11033-023-08756-z)
Supplement: Supplementary file 1 — Supplementary Material 1 [file 11033_2023_8756_MOESM1_ESM.docx]

**Molecular mechanism of paraquat-induced ferroptosis leading to pulmonary fibrosis mediated by Keap1/Nrf2 signaling pathway**

Xiaoxia Yang^1^, Ping Xiao^2^, Xiaofeng Shi^3*^

1 Department of Neurology, Tianjin First Central Hospital, Tianjin, 300192.

2 Clinical Laboratory, Tianjin First Central Hospital, Tianjin, 300192.

3 Department of Emergency, Tianjin First Central Hospital, Tianjin, 300192.

^*^Address correspondence to Xiaofeng Shi, E-mail: sxf74@sohu.com.

**Method S1. Transcriptomic analysis**

**1.1 RNA isolation and Illumina HiSeq sequencing**

Total RNA was extracted from each liver sample using TRIzol reagent according to the manufacturer’s protocol. RNA quality was assessed using the Agilent 2100 bioanalyzer (Agilent Technologies, Palo Alto, CA, USA), and the concentration was measured using the NanoDrop 1000 spectrophotometer (Thermo Fisher Scientific, Schwerte, Germany). For library preparation, 1 μg of total RNA with a RNA integrity number (RIN) > 7 was used. The NEBNext®Ultra™RNA Library Prep Kit for Illumina® was used following the manufacturer’s instructions. After library purification using Beckman agcourtAMPure XP microbeads, PCR products were cleaned using AxyPrep Mag PCR cleanup (axgen) and validated using the Agilent 2100 bioanalyzer. Quantification was performed using the Qubit 2.0 fluorometer (Invitrogen, Carlsbad, CA, USA). Libraries with different indexes were then multiplexed and loaded onto the Illumina HiSeq instrument (Illumina, San Diego, CA, USA) as per the manufacturer’s instructions. Sequencing was performed using the 2 × 150 bp terminal structure. Image analysis and base calling were conducted on the HiSeq instrument using HiSeq Control Software, Off-Line Basecaller, and GPipelin-1.6.

**1.2 Read filtering and mapping**

Reference genome sequence and gene model annotation files were downloaded from UCSC, NCBI, and ENSEMBL genome databases for the related species. The reference genome sequence was indexed using Hisat2 (v2.0.1). Subsequently, the clean data was aligned to the reference genome using Hisat2 (v2.0.1) software.

**1.3 Differential expression analysis and enrichment analysis of gene ontology (GO) and Kyoto Encyclopedia of Genes and Genomes (KEGG)**

Gene expression counts were measured using HTSeq (V0.6.1) based on transcript abundance. The gene expression levels were calculated using RPKM (Reads Per Kilobase per Million Reads). The read count was normalized with DESeq.2 (V1.6.3).

Differentially expressed genes (DEGs) were defined as having a fold change ≥ 2 and FDR ≤ 0.05. The gene ontology (GO) terms, which describe the cellular components, biological processes, and molecular functions of gene sets derived from differentially expressed genes (Huang et al., 2009), were annotated to enriched genes using Go-term Finder (V0.86) with a significance threshold of p<0.05.

KEGG, a comprehensive database of genomes, biological pathways, diseases, drugs, and chemicals (<http://en.wikipedia.org/wiki/KEGG>), was used for further analysis.

**Method S2. Proteomic analysis**

**2.1 Experiments**

**2.1.1 Protein extraction and digestion**

SDT(4%SDS，100mM Tris-HCl，1mM DTT，pH7.6) buffer was used for sample lysis and protein extraction. The amount of protein was quantified with the BCA Protein Assay Kit (Bio-Rad, USA). Protein digestion by trypsin was performed according to filter-aided sample preparation (FASP) procedure described by Matthias Mann. The digest peptides of each sample were desalted on C18 Cartridges (Empore™ SPE Cartridges C18 (standard density), bed I.D. 7 mm, volume 3 ml, Sigma), concentrated by vacuum centrifugation and reconstituted in 40 µl of 0.1% (v/v) formic acid.

**Filter-aided sample preparation (FASP Digestion)procedure**：200 μg of proteins for each sample were incorporated into 30 μl SDT buffer (4% SDS, 100 mM DTT, 150 mM Tris-HCl pH 8.0). The detergent, DTT and other low-molecular-weight components were removed using UA buffer (8 M Urea, 150 mM Tris-HCl pH 8.0) by repeated ultrafiltration (Microcon units, 10 kD). Then 100 μl iodoacetamide (100 mM IAA in UA buffer) was added to block reduced cysteine residues and the samples were incubated for 30 min in darkness. The filters were washed with 100 μl UA buffer three times and then 100 μl 25mM NH4HCO3 buffer twice. Finally, the protein suspensions were digested with 4 μg trypsin (Promega) in 40 μl 25mM NH4HCO3 buffer overnight at 37 °C, and the resulting peptides were collected as a filtrate. The peptides of each sample were desalted on C18 Cartridges (Empore™ SPE Cartridges C18 (standard density), bed I.D. 7 mm, volume 3 ml, Sigma), concentrated by vacuum centrifugation and reconstituted in 40 µl of 0.1% (v/v) formic acid. The peptide content was estimated by UV light spectral density at 280 nm using an extinctions coefficient of 1.1 of 0.1% (g/l) solution that was calculated on the basis of the frequency of tryptophan and tyrosine in vertebrate proteins.

**2.1.2 SDS-PAGE**

20 µg of protein for each sample were mixed with 5X loading buffer respectively and boiled for 5 min. The proteins were separated on 12.5% SDS-PAGE gel (constant current 14 mA, 90 min). Protein bands were visualized by Coomassie Blue R-250 staining.

**2.1.3****Labeling**

**iTRAQ：**100 μg peptide mixture of each sample was labeled using iTRAQ reagent according to the manufacturer’s instructions (Applied Biosystems).

**TMT ：**100 μg peptide mixture of each sample was labeled using TMT reagent according to the manufacturer’s instructions (Thermo Scientific).

**2.1.4 High pH Reversed-Phase Fractionation**

Labeled peptides were fractionated by High pH Reversed-Phase Peptide Fractionation Kit (Thermo Scientific). The dried peptide mixture was reconstituted and acidified with 0.1% TFA solution and loaded to the equilibrated, high-pH, reversed-phase fractionation spin column. Peptides are bound to the hydrophobic resin under aqueous conditions and desalted by washing the column with water by low-speed centrifugation. A step gradient of increasing acetonitrile concentrations in a volatile high-pH elution solution is then applied to the columns to elute bound peptides into 10 different fractions collected by centrifugation. The collected fractions were desalted on C18 Cartridges (Empore™ SPE Cartridges C18 (standard density), bed I.D. 7 mm, volume 3 ml, Sigma) and concentrated by vacuum centrifugation.

**2.1.5 LC-MS/MS analysis**

LC-MS/MS analysis was performed on a Q Exactive mass spectrometer (Thermo Scientific) that was coupled to Easy nLC (Proxeon Biosystems, now Thermo Fisher Scientific) for 60/90 min. The peptides were loaded onto a reverse phase trap column (Thermo Scientific Acclaim PepMap100, 100 μm*2 cm, nanoViper C18) connected to the C18-reversed phase analytical column (Thermo Scientific Easy Column, 10 cm long, 75 μm inner diameter, 3μm resin) in buffer A (0.1% Formic acid) and separated with a linear gradient of buffer B (84% acetonitrile and 0.1% Formic acid) at a flow rate of 300 nl/min controlled by IntelliFlow technology. The mass spectrometer was operated in positive ion mode. MS data was acquired using a data-dependent top10 method dynamically choosing the most abundant precursor ions from the survey scan (300–1800 m/z) for HCD fragmentation. Automatic gain control (AGC) target was set to 3e6, and maximum inject time to 10 ms. Dynamic exclusion duration was 40.0 s. Survey scans were acquired at a resolution of 70,000 at m/z 200 and resolution for HCD spectra was set to 17,500 at m/z 200, and isolation width was 2 m/z. Normalized collision energy was 30 eV and the underfill ratio, which specifies the minimum percentage of the target value likely to be reached at maximum fill time, was defined as 0.1%. The instrument was run with peptide recognition mode enabled.

**2.1.6 Identification and quantitation of proteins**

The MS raw data for each sample were searched using the MASCOT engine (Matrix Science, London, UK; version 2.2) embedded into Proteome Discoverer 1.4 software for identification and quantitation analysis.

**2.2 Bioinformatic analysis**

**2.2.1 Subcellular localization**

CELLO (<http://cello.life.nctu.edu.tw/>) which is a multi-class SVM classification system, was used to predict protein subcellular localization.

**2.2.2 Domain annotation**

Protein sequences are searched using the InterProScan software to identify protein domain signatures from the InterPro member database Pfam.

**2.2.3 GO annotation**

The protein sequences of the selected differentially expressed proteins were locally searched using the NCBI BLAST+ client software (ncbi-blast-2.2.28+-win32.exe) and InterProScan to find homologue sequences, then gene ontology (GO) terms were mapped and sequences were annotated using the software program Blast2GO. The GO annotation results were plotted by R scripts.

**2.2.4 KEGG annotation**

Following annotation steps, the studied proteins were blasted against the online Kyoto Encyclopedia of Genes and Genomes (KEGG) database (http://geneontology.org/) to retrieve their KEGG orthology identifications and were subsequently mapped to pathways in KEGG.

**2.2.5 Enrichment analysis**

Enrichment analysis were applied based on the Fisher’ exact test, considering the whole quantified proteins as background dataset. Benjamini- Hochberg correction for multiple testing was further applied to adjust derived p-values. And only functional categories and pathways with p-values under a threshold of 0.05 were considered as significant.

**Table S1.**Primers sequence used in real-time PCR experiments

| **Primers** | **Forward sequences (5’-3’)** | **Reverse sequences (5’-3’)** |
| --- | --- | --- |
| *GPX4* | GAGGCAGGAGCCAGGAAGTAATC | CACGCAGCCGTTCTTATCAATG |
| *FTH1* | TGCCAAATACTTTCTCCATCAATCTC | CCAGTCATCACGGTCAGGTTTC |
| *TFR1* | AGACTCTGCTTTGCGACTATTGC | CACACACTCCTCTTTTTGTTCTACG |
| *DMT1* | CCTGAAGAAAAGATTCCAGACGATG | TCAAAGTAGGTGGTGAAGGGCTC |
| *FTL* | GAAGATGGGCAACCACCTGACC | TAGTCGTGCTTCAGAGTGAGGCG |
| *Nrf2* | GGCGGGAGGACCTTCTGTATGC | GGCCCAATTTTGTTCCACCTCTCC |
| *Nqo1* | AGAAACGACATCACAGGGGAGC | ACAAGCACTCTCTCAAACCAGCC |
| *GAPDH* | TTCAGCTCTGGGATGACCTT | TGCCACTCAGAAGACTGTGG |





**Figure S1.**Lung quotiety (n=6).


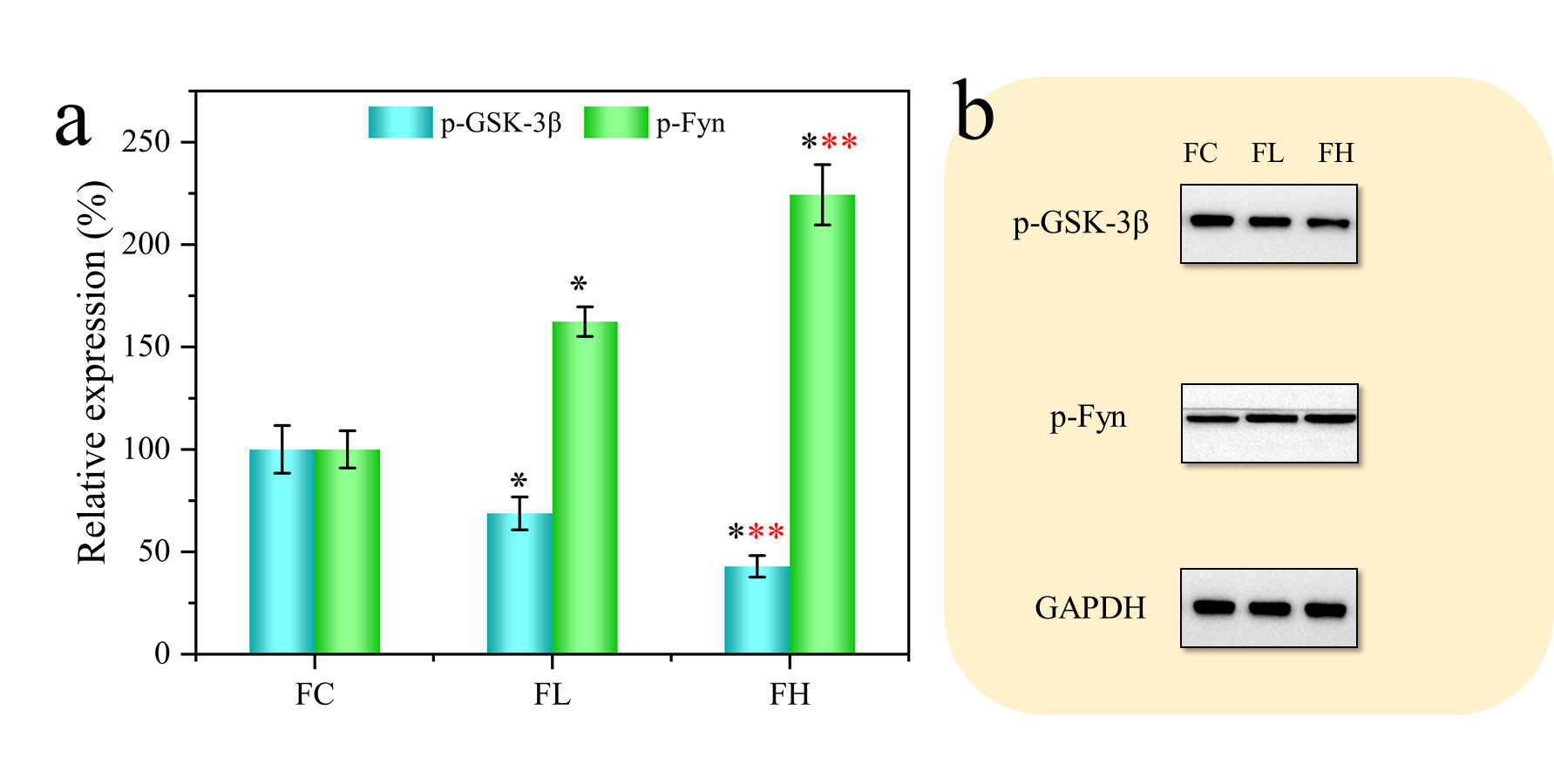


**Figure S2.** Western Blotting analysis of p-GSK-3β and p-Fyn and their WB bands (n=6).


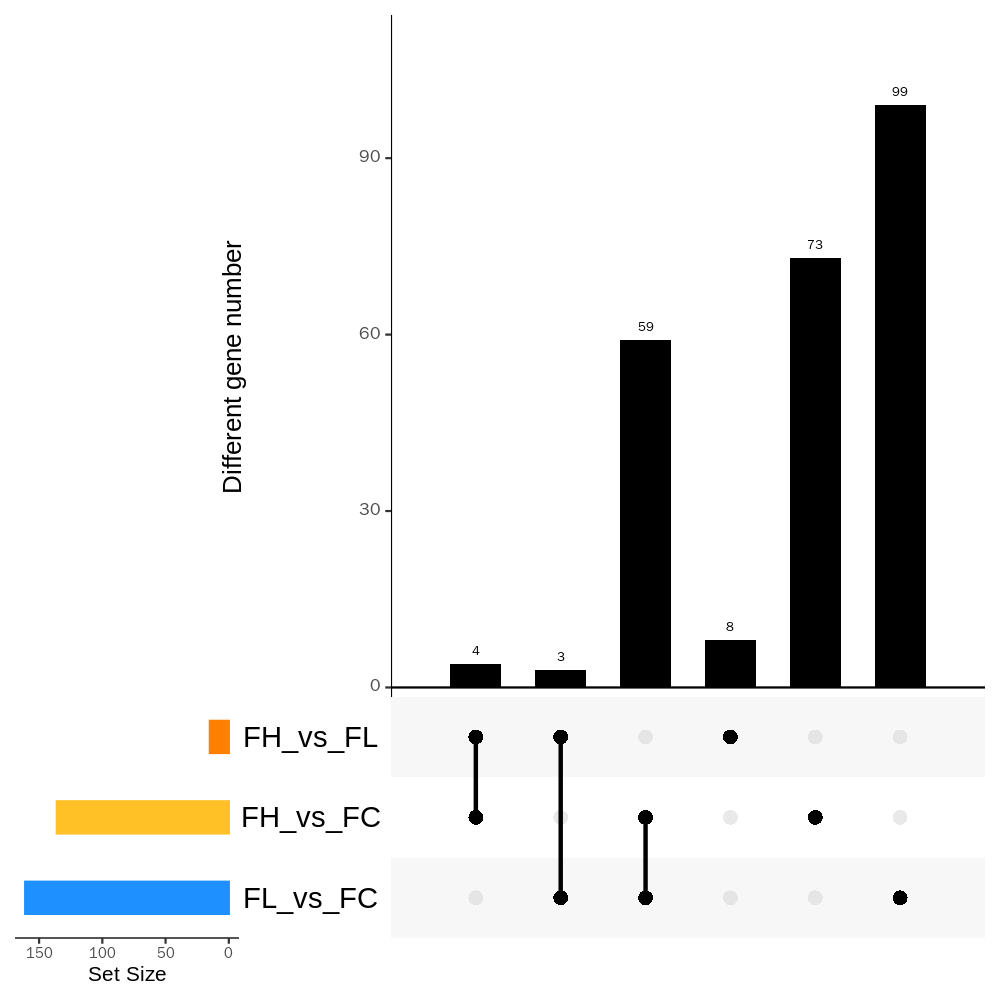


**FigureS3.**Statistics of different genes between groups.


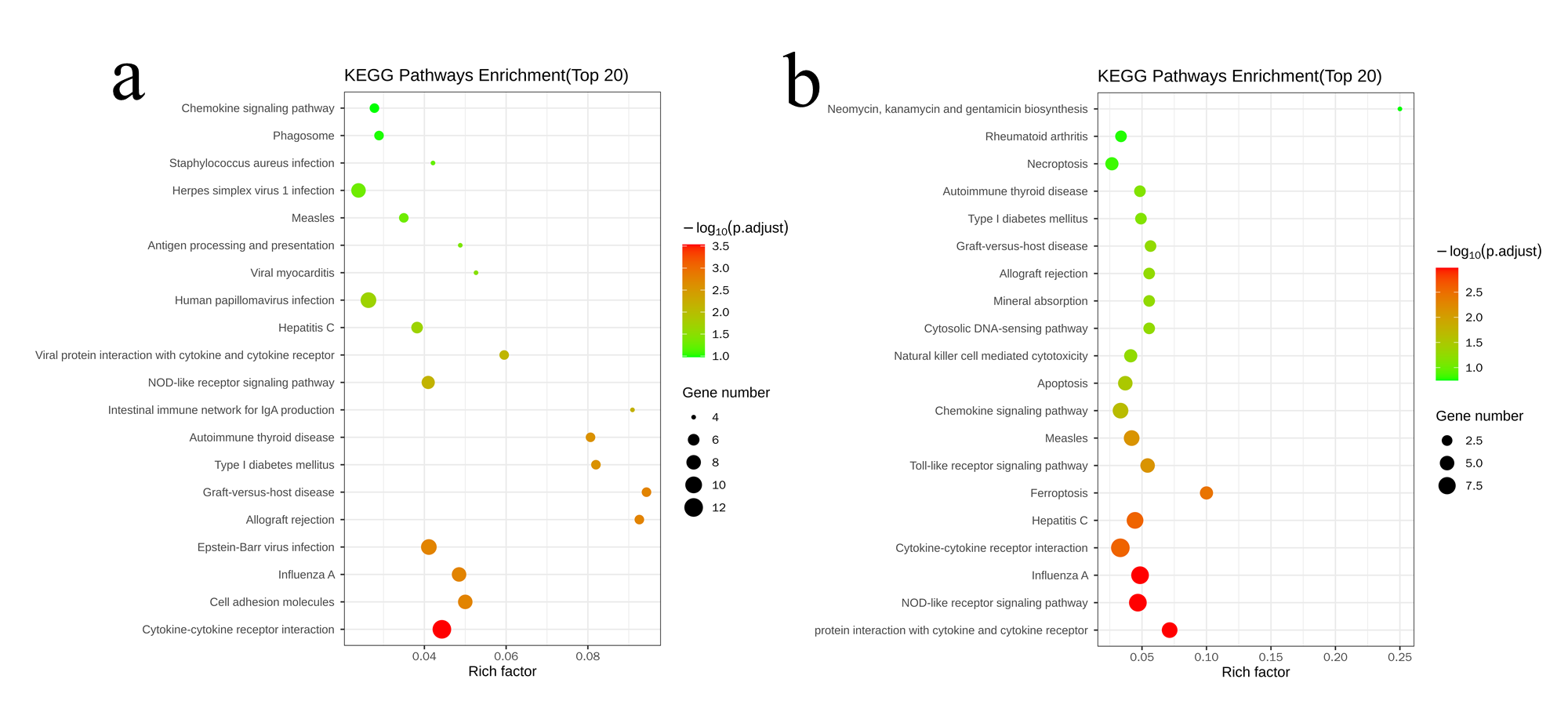


**Figure S4.**Intergroup enrichment of KEGG pathways (a) FL vs FC, (b) FH vs FC.


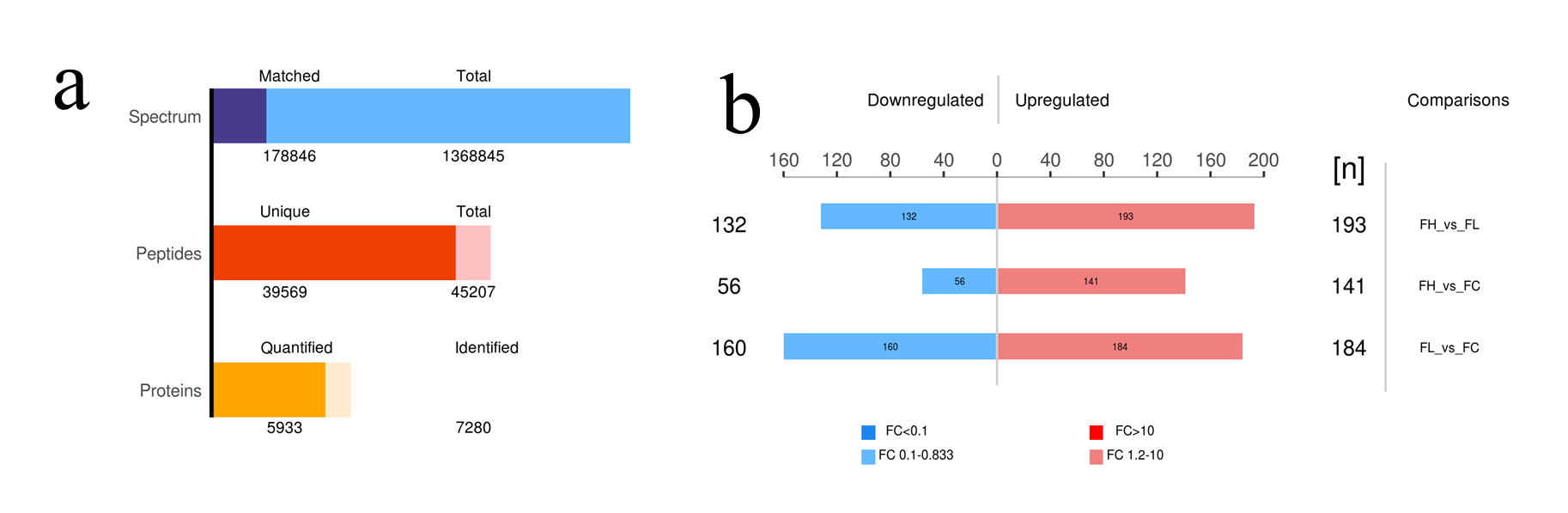


**Figure S5.**(a) The total number of matched proteins and (b) the number of different proteins between groups.


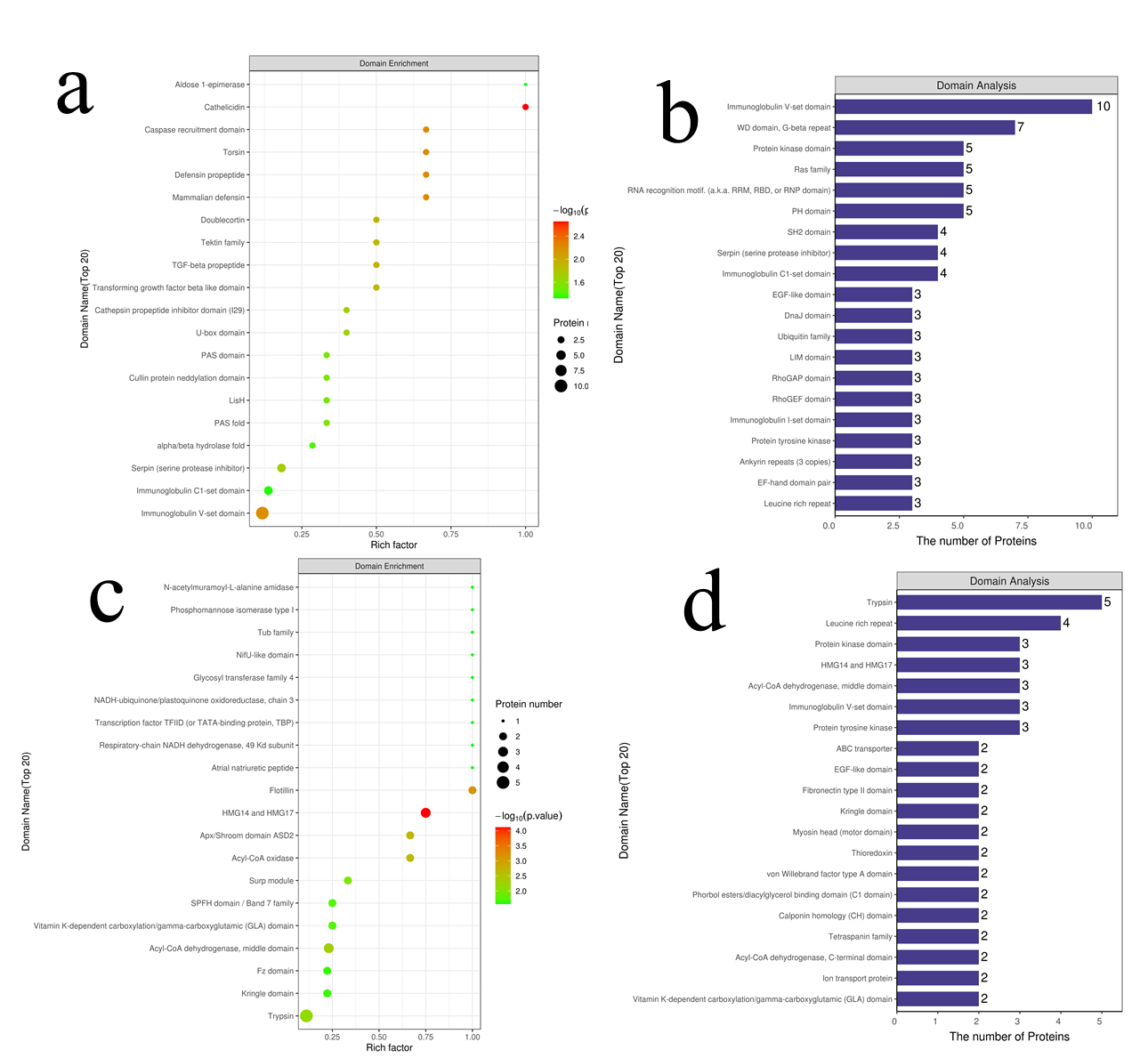


**Figure S6.**Effects of PQ exposure on protein domains. (a) enrichment analysis and (b) composition statistics of the domain between FL and FC. (c) enrichment analysis and (d) composition statistics of the domains between FH and FC.
